# Growing attention on the toxicity of Chinese herbal medicine: a bibliometric analysis from 2013 to 2022

**DOI:** 10.3389/fphar.2024.1293468

**Published:** 2024-02-01

**Authors:** Ke-Xin Zhu, Min Wu, Zhi-Lin Bian, Shi-Liang Han, Li-Ming Fang, Feng-Feng Ge, Xue-Zhou Wang, Sheng-Fang Xie

**Affiliations:** ^1^ Affiliated Hospital of Integrated Traditional Chinese and Western Medicine, Nanjing University of Chinese Medicine, Nanjing, China; ^2^ Jiangsu Province Academy of Traditional Chinese Medicine, Nanjing, China; ^3^ International Acupuncture and Moxibustion Innovation Institute, School of Acupuncture-Moxibustion and Tuina, Beijing University of Chinese Medicine, Beijing, China

**Keywords:** Chinese herbal medicine, toxicity, safety, bibliometric analysis, crosssectional, pharmacology

## Abstract

**Introduction:** Despite the clinical value of Chinese herbal medicine (CHM), restricted comprehension of its toxicity limits the secure and efficacious application. Previous studies primarily focused on exploring specific toxicities within CHM, without providing an overview of CHM’s toxicity. The absence of a quantitative assessment of focal points renders the future research trajectory ambiguous. Therefore, this study aimed to reveal research trends and areas of concern for the past decade.

**Methods:** A cross-sectional study was conducted on publications related to CHM and toxicity over the past decade from Web of Science Core Collection database. The characteristics of the publication included publication year, journal, institution, funding, keywords, and citation counts were recorded. Co-occurrence analysis and trend topic analysis based on bibliometric analysis were conducted on keywords and citations.

**Results:** A total of 3,225 publications were analyzed. Number of annal publications increased over the years, with the highest number observed in 2022 (n = 475). The *Journal of Ethnopharmacology* published the most publications (n = 425). The most frequently used toxicity classifications in keywords were hepatotoxicity (n = 119) or drug-induced liver injury (n = 48), and nephrotoxicity (n = 40). Co-occurrence analysis revealed relatively loose connections between CHM and toxicity, and their derivatives. Keywords emerging from trend topic analysis for the past 3 years (2019–2022) included ferroptosis, NLRP3 inflammasome, machine learning, network pharmacology, traditional uses, and pharmacology.

**Conclusion:** Concerns about the toxicity of CHM have increased in the past decade. However, there remains insufficient studies that directly explore the intersection of CHM and toxicity. Hepatotoxicity and nephrotoxicity, as the most concerned toxicity classifications associated with CHM, warrant more in-depth investigations. Apoptosis was the most concerned toxicological mechanism. As a recent increase in attention, exploring the mechanisms of ferroptosis in nephrotoxicity and NLRP3 inflammasome in hepatotoxicity could provide valuable insights. Machine learning and network pharmacology are potential methods for future studies.

## 1 Introduction

Herbal medicine is derived from traditional medical systems and serves as an extensively employed complementary therapy in clinical practice (Barnes et al., 2016). Particularly, Chinese herbal medicine (CHM) stands as a representative within this domain, boasting a long historical lineage and even contributed China’s first Nobel Prize in natural sciences (Tu, 2016). Evidence supports the protentional efficacy of CHM in treating cardiovascular (Hao et al., 2017), nephritic (Zhong et al., 2015), immunologic (Jakobsson et al., 2022) and cancer-related disorders (Yao et al., 2021). As one of the most prevalent herbal medicines, CHM is exported to over 175 countries and regions (Teng et al., 2016). Despite these promising prospects, similar to other herbal remedies, CHM encounters the hurdle of toxicity. The toxicity of CHM typically presents as hepatotoxicity and nephrotoxicity (Ekor, 2014), which are the leading causes of drug development failure or market withdrawal (Hoppmann et al., 2020; Kulkarni, 2021). Given the World Health Organization’s prioritization of drug safety (Donaldson et al., 2017), emphasizing the toxicity of CHM becomes imperative.

The underestimation of the toxicity of CHM is a longstanding issue. The prescription of CHM under the guidance of traditional toxicity theory is frequently considered to be completely safe. Following the first discovery of severe toxic aristolochic acids in some Chinese herbs (Vanhaelen et al., 1994), many toxic components were determined (Lv et al., 2012). However, before the prohibition of these CHMs and synthetic drugs by countries (Hashimoto et al., 1999; Lee et al., 2002; Gabardi et al., 2007; Zhou et al., 2013), the associated herbs had been used for thousands of years. Astonishingly, the modern toxicity of some CHMs contradicts the traditional theory. For instance, *Aristolochia manshuriensis* Kom was traditionally believed to possess diuretic properties for treating kidney disease but was proven to be nephrotoxic and carcinogenic (Wang L. et al., 2018). Recent studies revealed that the toxicity of certain CHMs were quite complex, particularly in terms of hepatotoxicity. The toxicity of these herbs exhibits a dose-independent pattern and displays significant inter-individual variability across different populations (Ma et al., 2023). In addition, the lack of quality control measures during production and sales exacerbates the risks associated with toxicity (Raynor et al., 2011; Liu et al., 2015). Such concerns prompted regulatory bodies including the Food and Drug Administration (FDA) to only approve limited CHMs (Qu et al., 2022; You et al., 2022).

Fortunately, the toxicity of CHM garnered increased attention in recent years, with various safety regulatory standards, clinical practice guidelines and manifestos highlighting the urgency of this issue (Yang et al., 2022). However, existing studies mainly focused on specific toxicities, resulting in a lack of overview to the overall safety of CHM. Additionally, there are still gaps in understanding the actual incidence rate and specific mechanisms (Teschke et al., 2014; Yang et al., 2018; Wang et al., 2022). Therefore, to provide a comprehensive understanding of the toxicity of CHM and identify potential avenues for future research, this cross-sectional study aimed to reveal research trends and areas of concern for the past decade through bibliometric analysis.

## 2 Materials and methods

### 2.1 Data collection

This study included publications concerning the toxicity of CHM, and were published between 2013 and 2022. The search was conducted by the Web of Science Core Collection (WOS) database using the following strategy:

#1 TS= “toxicity” OR “toxicology” OR “nephrotoxicity” OR “hepatotoxicity” OR “drug-induced injury” OR “cardiotoxicity” OR “neurotoxicity” OR “ototoxicity” OR “hematotoxicity” OR “immunotoxicity”.

#2 TS= “traditional Chinese medicine” OR “Chinese herbal” OR “Chinese herb”.

#3 FPY = 2013–2022.

#1 AND #2 AND #3.

An additional search was conducted within the CNKI database to examine the distinctions in publication characteristics across databases. The search strategy used for the CNKI database is provided in the Supplementary Material.

### 2.2 Analysis

After obtaining the data of all publications, basic characteristics including title, publication year, author, author’s keyword, institution, funding agency and citation were counted. VOSviewer (version 1.6.19), Bibliometrix package of R (version 4.3.0) and Citespace (version 5.7. R5) software were used for visualized analysis (van Eck and Waltman, 2010). Bibliographic coupling analysis with timeline was performed in influential journals. Influential journals were defined as the top 25 journals (≥5 publications on this field per journal) with the most cite frequency by the included publications. Evaluation of hotspot and trend was conducted by co-occurrence analysis and trend topic analysis based on author’s keywords. Co-occurrence analysis was conducted on the 40 keywords of the most records, with varied colors showing the trends over time. Additional co-occurrence analysis was conducted on countries with more than 10 publications for exploring the cooperation between individual countries. Trend topic analysis was conducted on the 3 independent emerging keywords each year, with the duration of the hot topic. In trend topic analysis, keywords lasted for more than 5 years were defined as long-term concerned keywords, while the ones with the duration less than 2 years were defined as short-term concerned keywords. Co-occurrence analysis and trend topic analysis are complementary. The former selects the term of analysis by keyword frequency, which can show the connection between keywords, but may mask the recent emerging keywords. Trend topic analysis does not have the ability to show connections, but can better detect the potential hotspots. Citation burst analysis was conducted to discern emergent citations.

## 3 Results

The initial search yielded 3,242 records, and 17 of them were excluded for reasons including languages other than English (n = 12), retracted publication (n = 3) and correction (n = 2). A total of 3,225 publications were included in the analysis, with 2,413 articles, 785 reviews, 12 meeting abstracts, 11 editorial materials and 4 letters.

### 3.1 Basic characteristics

**FIGURE 1:**
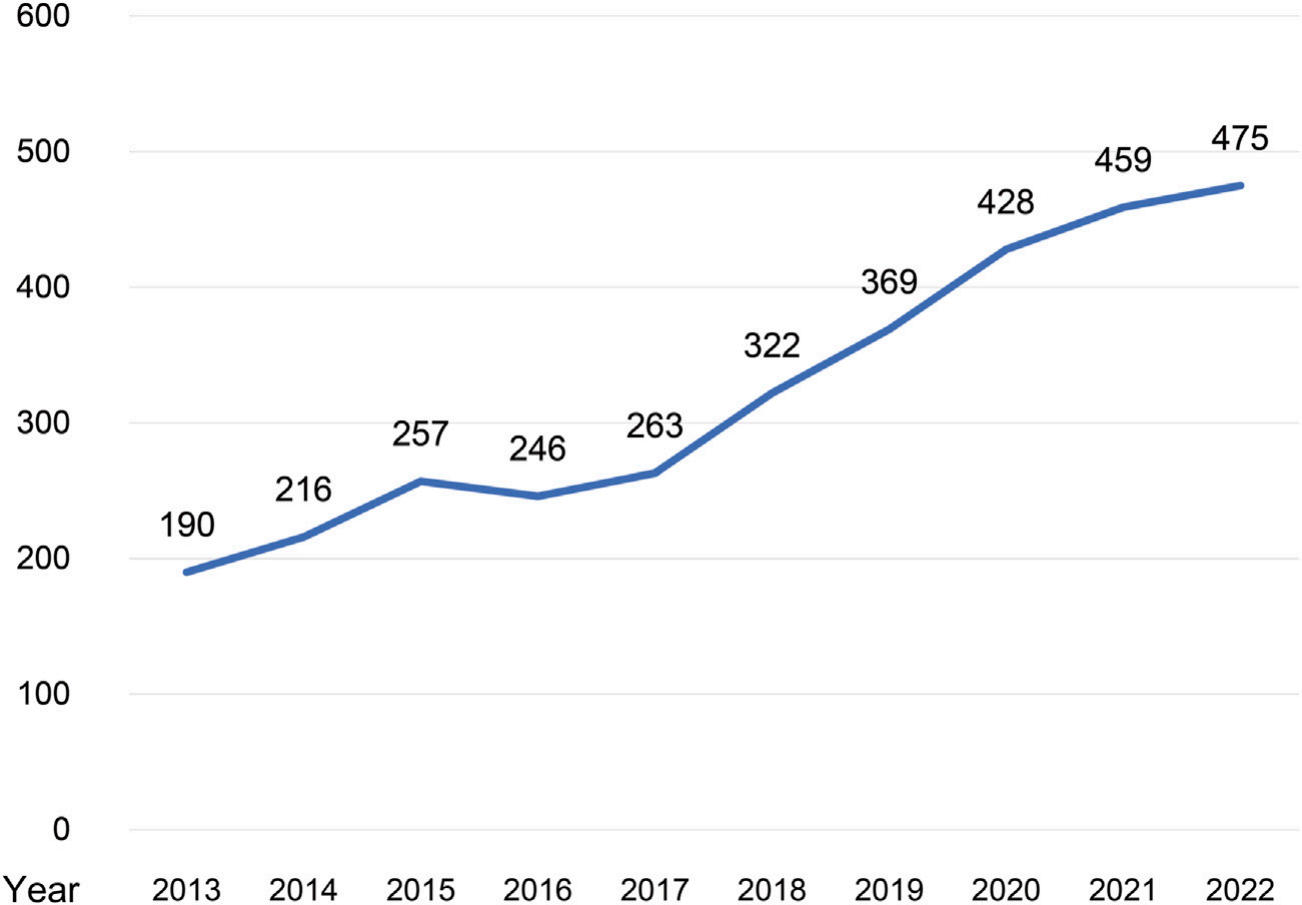
Number of annal publications on the toxicity of Chinese herbal medicine.

**FIGURE 2:**
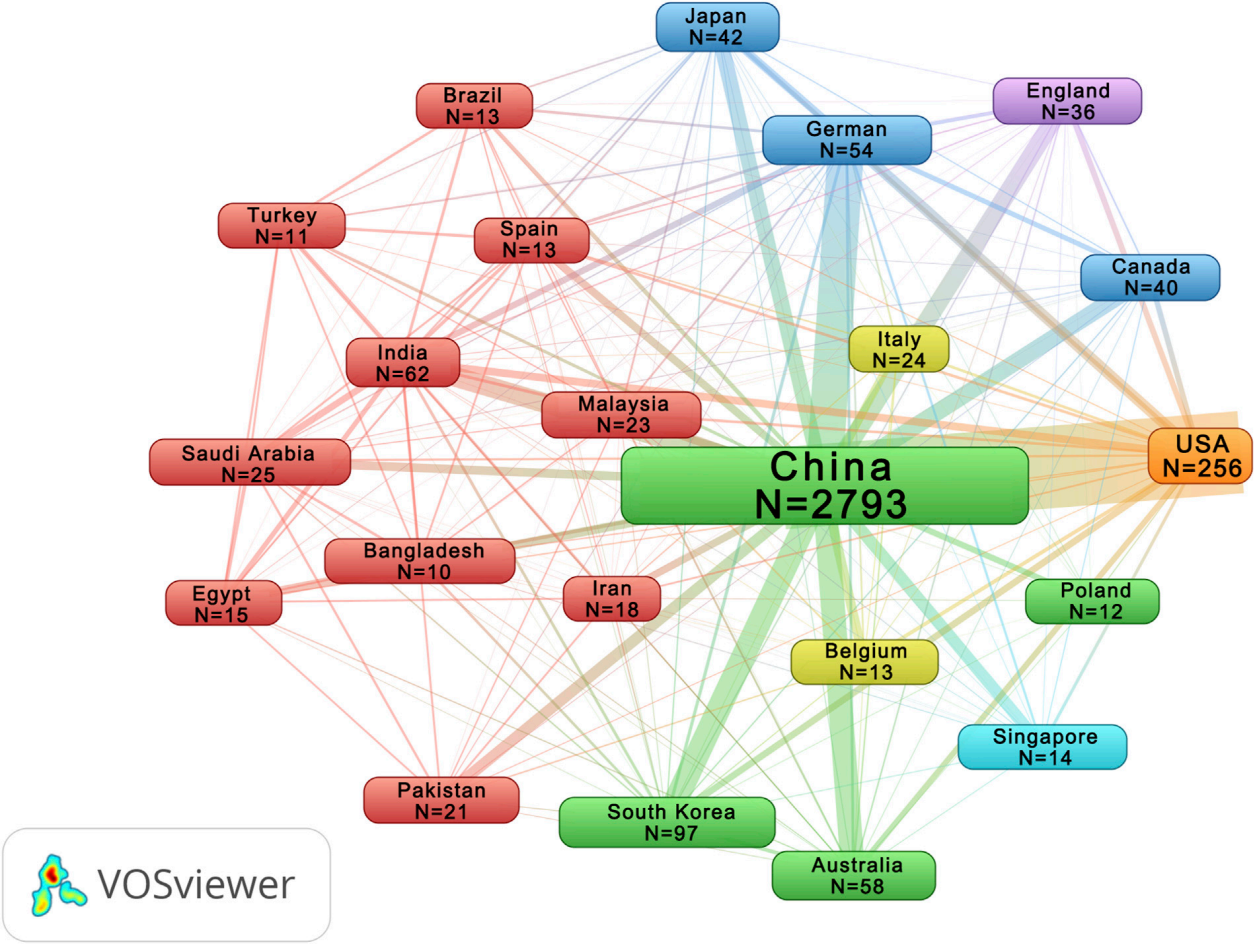
Co-occurrence analysis of countries. Countries with more than 10 publications were included. N indicates the number of publications. The thickness of the lines in the visualization indicates the frequency of co-occurrence between elements.

From 2013 to 2022, with the exception of a slight decline in 2016 (n = 246), annual publications were increased by the year (Figure 1). The number of publications reached its highest in 2022 (n = 475). As showing in Table 1, the top 5 productive journals in this field were *Journal of Ethnopharmacology* (n = 425), *Frontiers in Pharmacology* (n = 226), *Evidence-Based Complementary and Alternative Medicine* (n = 138), *Molecules* and *Phytomedicine* (n = 76). Beijing University of Chinese Medicine (n = 187) contributed the most publications. National Natural Science Foundation of China (n = 1,489) is the agency that supported the most publications in this field. The top 5 institutions and funding agencies were all Chinese. Table 2 displays the leading 5 authors alongside their respective h-index values, representing the individuals with the highest number of publications. Xiao-He, Xiao had the most publications and the highest h-index among the authors listed. Figure 2 delineates the publication volume and cooperative connections among countries. China stands out as the most prolific region (n = 2,793), exhibiting collaboration primarily with the United States and Germany.

**TABLE 1:** Top 5 productive journals, institutions and funding agencies.

Category		Counts (%)
Journal	Journal of Ethnopharmacology (IF = 5.4, Q1)^a^	425 (13.18)
	Frontiers in Pharmacology (IF = 5.6, Q1)	226 (7.01)
	Evidence-Based Complementary and Alternative Medicine^b^	138 (4.28)
	Molecules (IF = 4.6, Q2)	77 (2.39)
	Phytomedicine (IF = 7.9, Q1)	76 (2.36)
Institution	Beijing University of Chinese Medicine	187 (5.80)
	Chengdu University of Traditional Chinese Medicine	152 (4.71)
	Shanghai University of Traditional Chinese Medicine	146 (4.52)
	Nanjing University of Chinese Medicine	132 (4.09)
	Chinese Academy of Medical Sciences and Peking Union Medical College	118 (3.66)
Funding agency	National Natural Science Foundation of China	1,489 (46.17)
	China Postdoctoral Science Foundation	87 (2.70)
	National Basic Research Program of China	78 (2.42)
	Fundamental Research Funds for the Central Universities	74 (2.29)
	National Natural Science Foundation of Guangdong Province	65 (2.02)

^a^ Impact factor (IF) and quartile ranking were based on the Journal Citation Reports (JCR) data updates 18 Oct 2023.

^b^ The journal no longer includes in Web of Science (WOS) journal list since 20 March 2023.

**TABLE 2:** Top 5 productive authors.

Author	Counts^a^	H-index^b^
Xiao-He, Xiao	30	87
Jia-Bo, Wang^c^	25	36
Jin-Ao, Duan	24	51
Yu-Ping, Tang	22	51
Tomas, Efferth	17	79

^a^ Due to the duplication of names, publications were counted by individual authors, rather than authors’ name. After formatting all names manually, authors’ information was verified in WOS to prevent mismatching.

^b^ The h-index is an indicator based on a list of publications ranked in descending order by the Times Cited and reflects the productivity of authors based on their publication and citation records. H-index of the authors were recorded on 25 November 2023.

^c^ Two author profiles appeared in WOS, that were verified to belong to the same individual author. Publications were combined counted and h-index was selected from the profile with more publications.

### 3.2 Most cited publications and journals

**FIGURE 3:**
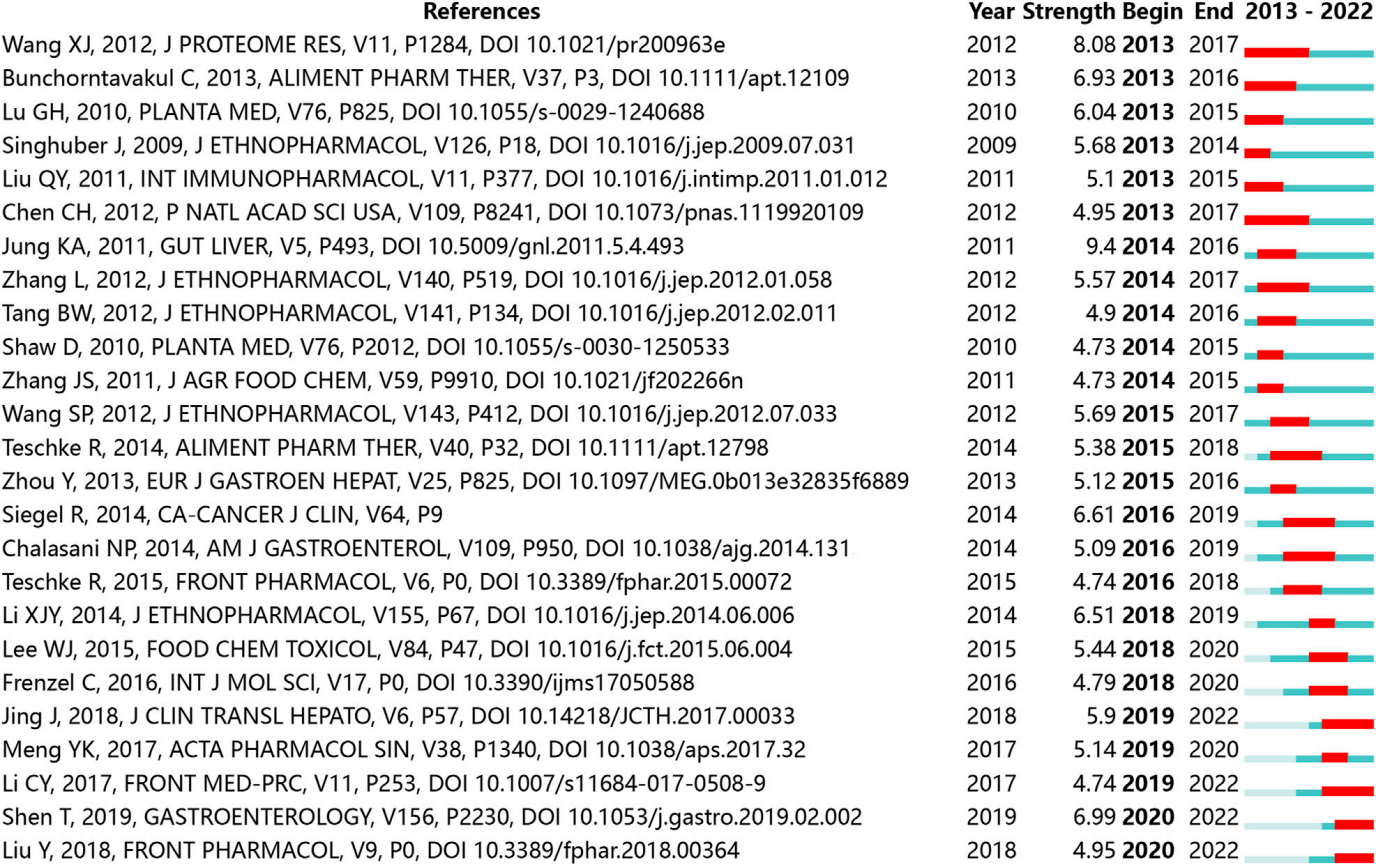
Top 25 citations with the strongest bursts.

**FIGURE 4:**
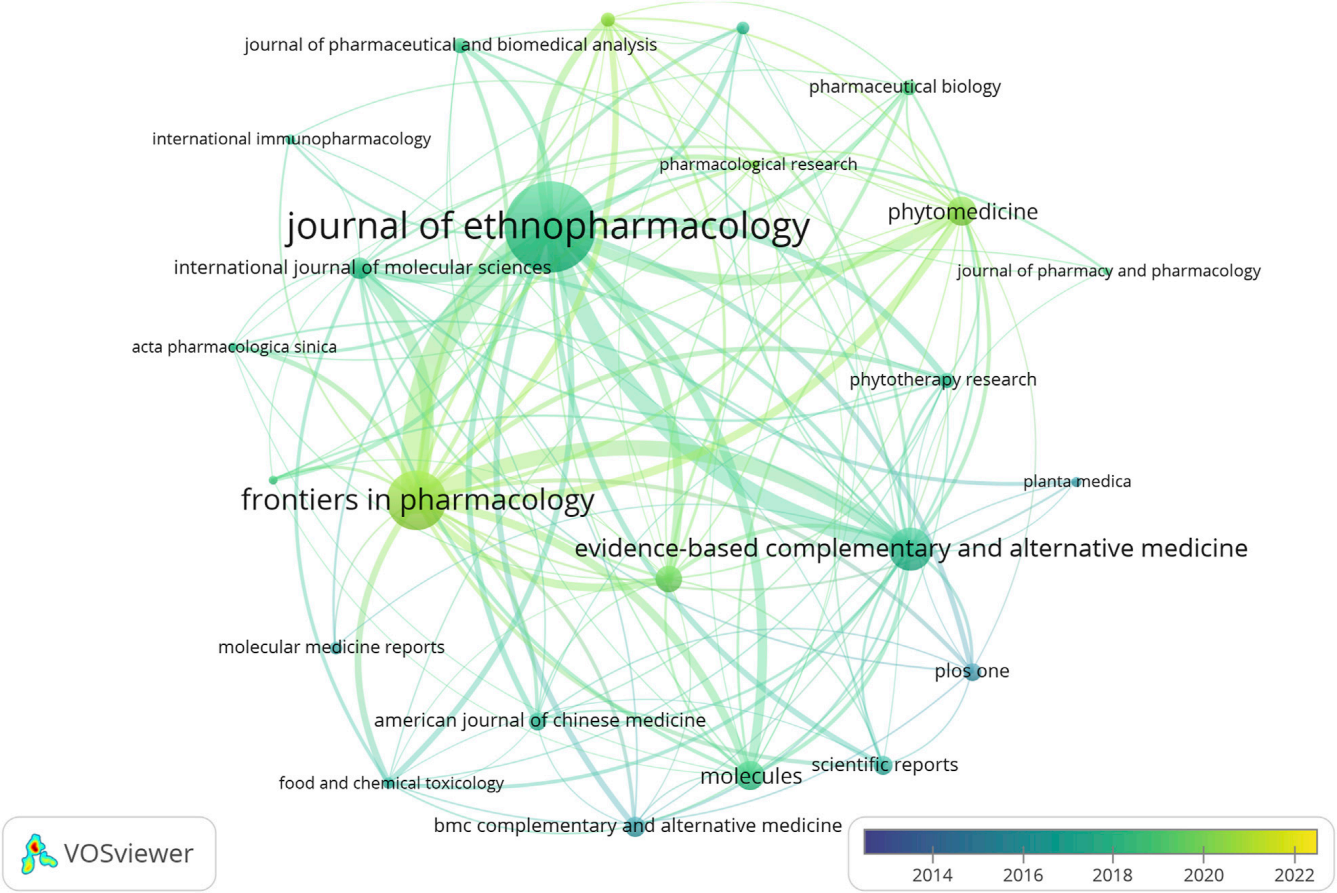
Coupling analysis of 25 influential journals. The thickness of the lines in the visualization indicates the frequency of co-occurrence between elements.

Table 3 lists the 10 most cited publications, with 9 reviews and 1 research article. The publication entitled “The growing use of herbal medicines: issues relating to adverse reactions and challenges in monitoring safety (Ekor, 2014)” got the most citations (1,416 citations). Within these 10 publications, 8 were contributed by Chinese researches, 5 were published on the aforenamed top 5 journals. None was published on multidisciplinary journals. Figure 3 shows the temporal burst of cited publications over the last decade in the included publications. “Screening for main components associated with the idiosyncratic hepatotoxicity of a tonic herb, Polygonum multiflorum (Li et al., 2017)”, “Incidence and Etiology of Drug-Induced Liver Injury in Mainland China (Shen et al., 2019)” and “Polygonum multiflorum Thunb.: A Review on Chemical Analysis, Processing Mechanism, Quality Evaluation, and Hepatotoxicity (Liu et al., 2018)” were emerged as focal points, garnering significant citations in recent years. The top 5 frequently cited journals by the included publications were *Journal of Ethnopharmacology* (n = 13,159), *Frontiers in Pharmacology* (n = 5,099), *Molecules* (n = 1,503), *Evidence-Based Complementary and Alternative Medicine* (n = 1,489), *International Journal of Molecular Sciences* (n = 1,347). Figure 4 shows the coupling result of the influential journals. The patterns of journal citations transferred over time, with a recent surge in the popularity of citing journals such as *Frontiers in Pharmacology* and *Phytomedicine*.

**TABLE 3:** Top 10 publications with the most citations.

Title	Corresponding author^a^	Journal	Publication year	Cited frequency^b^
The growing use of herbal medicines: issues relating to adverse reactions and challenges in monitoring safety	Ekor, M	Frontiers in Pharmacology	2014	1,416
Network Pharmacology Databases for Traditional Chinese Medicine: Review and Assessment	Ning, K	Frontiers in Pharmacology	2019	530
Puerarin: A Review of Pharmacological Effects	Peng, C	Phytotherapy Research	2014	427
Emodin: A Review of its Pharmacology, Toxicity and Pharmacokinetics	Ni, J	Phytotherapy Research	2016	364
From ancient herb to modern drug: Artemisia annua and artemisinin for cancer therapy	Efferth, T	Seminars in Cancer Biology	2017	317
Apigenin in cancer therapy: anti-cancer effects and mechanisms of action	Shao, HJ	Cell and Bioscience	2017	267
Triptolide: Progress on research in pharmacodynamics and toxicology	Jiang, ZZ	Journal of Ethnopharmacology	2014	255
Traditional uses, botany, phytochemistry, pharmacology and toxicology of Panax notoginseng (Burk.) FH Chen: A review	Wang, ZJ	Journal of Ethnopharmacology	2016	254
Incidence and Etiology of Drug-Induced Liver Injury in Mainland China	Mao, YM	Gastroenterology	2019	244
Traditional usages, botany, phytochemistry, pharmacology and toxicology of Polygonum multiflorum Thunb.: A review	Ni, J	Journal of Ethnopharmacology	2015	239

^a^ When a publication contains multiple corresponding authors, only the last one is presented.

^b^ Cited frequency was counted on 13 July 2023.

### 3.3 Hotspots and trends

Table 4 lists the top 25 keywords with the most frequencies. In terms of toxic classifications, hepatotoxicity (n = 119) or drug-induced liver injury (n = 48), and nephrotoxicity (n = 40) were focused on. In physiological and pathological manifestations, apoptosis (n = 190), oxidative stress (n = 115), inflammation (n = 81) and autophagy (n = 66) were focused on. In methods, pharmacokinetics (n = 93), metabolomics (n = 88), network pharmacology (n = 71), meta-analysis (n = 42) received great attentions.

**TABLE 4:** Top 25 keywords with the most frequencies.

Keyword	Count
Traditional Chinese medicine	263
Apoptosis	190
Pharmacology	168
Toxicity	167
Phytochemistry	143
Hepatotoxicity	119
Oxidative stress	115
Pharmacokinetics	93
Toxicology	93
Metabolomics	88
Inflammation	81
Alzheimer’s disease	79
Chinese herbal medicine	75
Network pharmacology	71
Autophagy	66
Traditional uses	56
Triptolide	53
Drug-induced liver injury	48
Herbal medicine	47
Safety	47
Chemotherapy	45
Neuroprotection	43
Meta-analysis	42
Nephrotoxicity	40
Review	39

**FIGURE 5:**
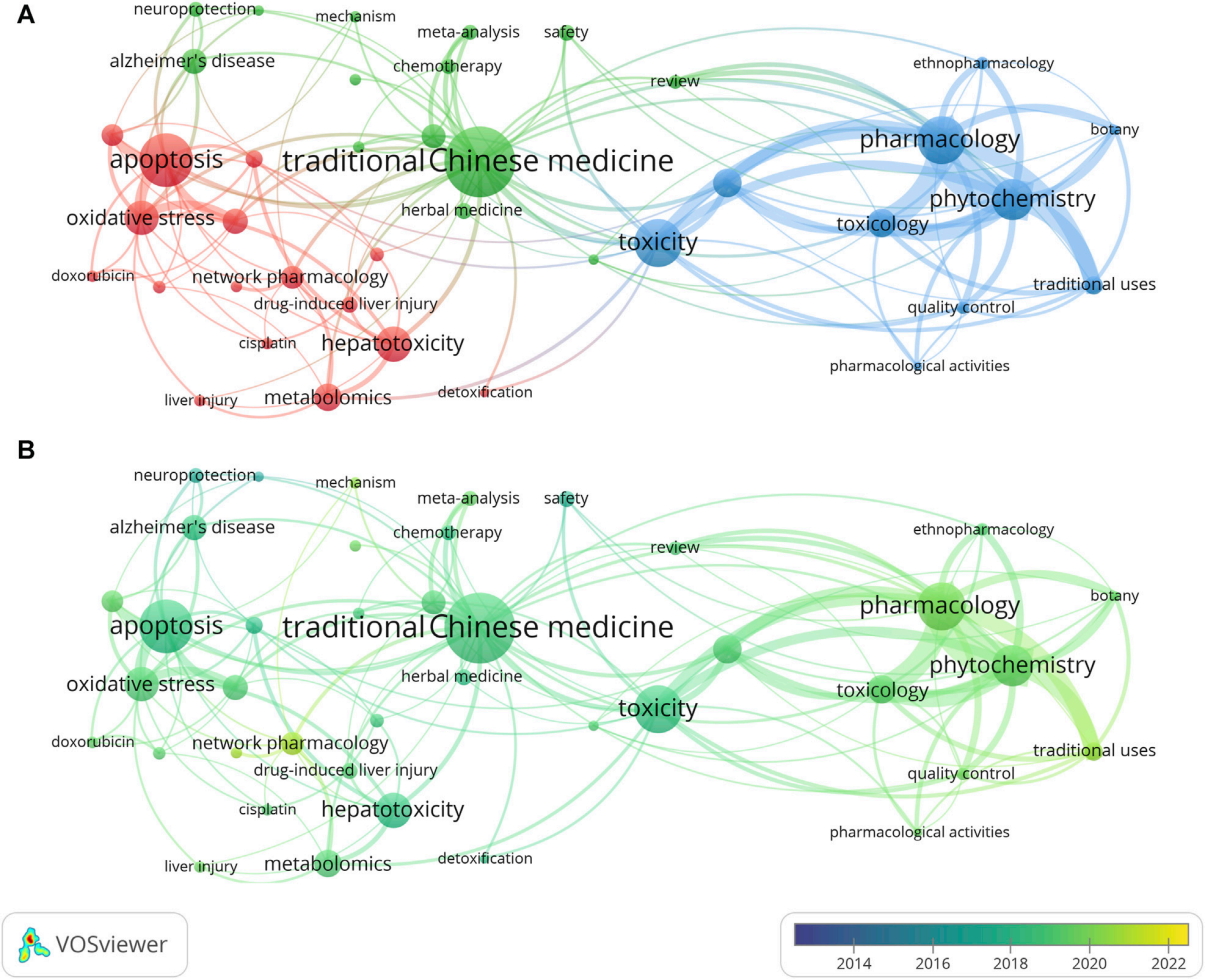
Co-occurrence analysis on the 40 keywords of the most records **(A)**, clustering result; **(B)**, clustering result with time-variation). The thickness of the lines in the visualization indicates the frequency of co-occurrence between elements.

After co-occurrence clustering of the top 40 keywords, 3 clusters were determined (Figure 5A). The keywords contained in cluster 1 (green cluster) were mainly CHM and its derivative words, which is characterized by the weak connection in-cluster and between-clusters. Cluster 2 (red cluster) contained the most keywords (n = 19). Among the most frequent words, apoptosis often co-occurred with oxidative stress and autophagy. Keywords in cluster 3 (blue cluster) were closely connected. Toxicity and toxicology often co-occurred with pharmacology or phytochemistry. The two keywords with the strongest correlation were pharmacology and phytochemistry. Figure 5B shows the change of keyword co-occurrence over time. Co-occurring keywords tended to shift from Cluster 1 and Cluster 2 to Cluster 3 between 2020 and 2022.

**FIGURE 6:**
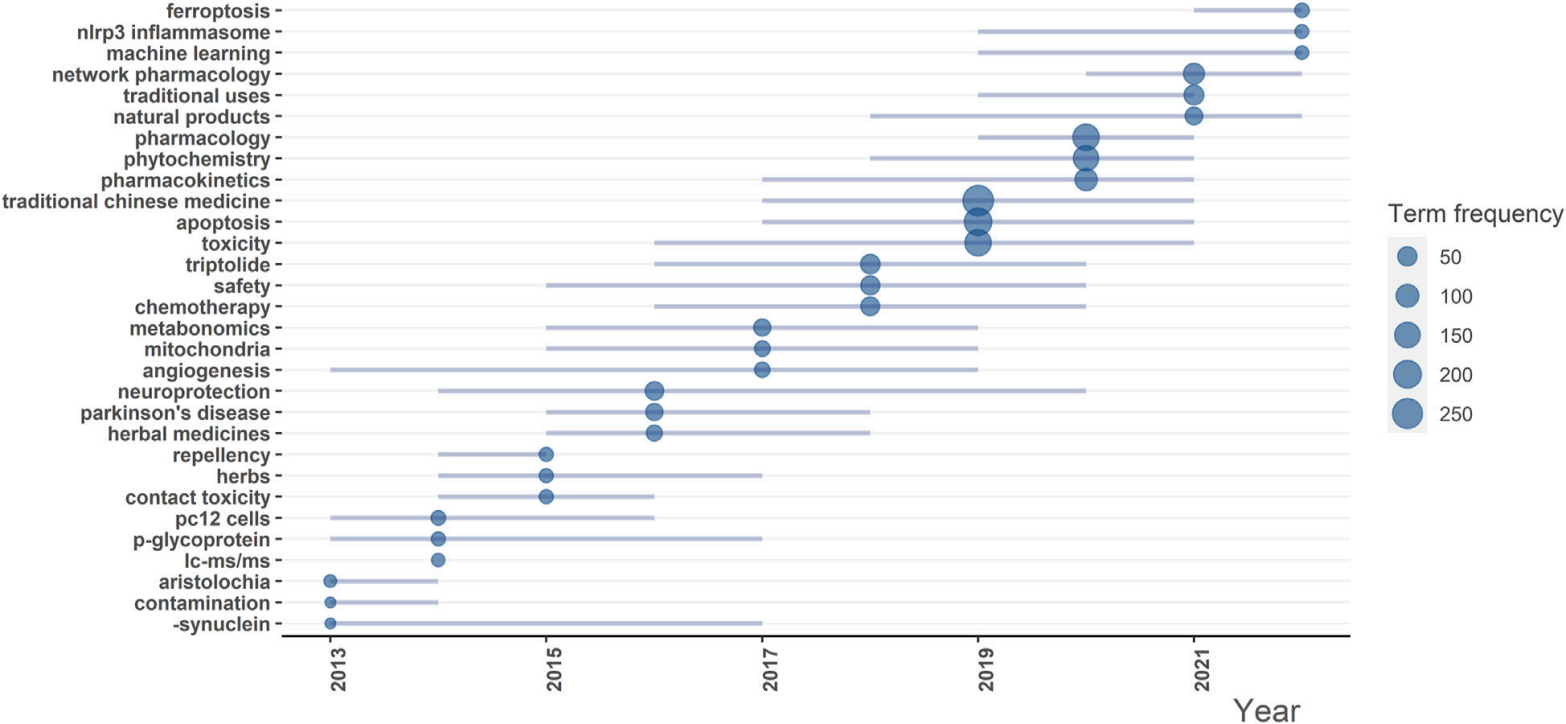
Trend topic analysis. The blue line signifies the duration of the keyword’s status as a hot topic, while the size of the blue dot indicates the frequency.

Figure 6 presents the results of trend topic analysis. Emerging keywords for the past 3 years (2019–2022) included ferroptosis, NLRP3 inflammasome, machine learning, network pharmacology, traditional uses and pharmacology. Long-term concerned keywords (>5 years) included toxicity, safety, angiogenesis and neuroprotection. Short-term concerned keywords (<2 years) included ferroptosis, repellency, LC-MS/MS, aristolochia and contamination.

### 3.4 Features of publications in the CNKI database

The search in CNKI database generated 5,880 records, comprising 3,306 journal articles, 1894 dissertations, 465 conference articles and 215 other types of publications. Supplementary Figure S1 and Supplementary Table S1 show the frequencies of the top 40 keywords and their co-occurrence network. A substantial portion of the top 40 words exhibited overlap, often conveying similar concepts. In contrast to the WOS database, publications within the CNKI database paid extra attention to the issues in CHM production process, while displaying less attention to the mechanism of CHM toxicity.

## 4 Discussion

Despite concerns about the toxicity of CHM, its social hazards persist. There is a misconception that CHM is non-toxic and universally applicable. Hence, adverse events caused by the toxicity of CHM occurs in both clinical practice and the public health field. Notably, China experiences a higher proportion of toxic incidents caused by herbs compared to other countries (Yang et al., 2022). As one of the oldest herbal remedies, CHM is often overused outside of prescription. In Asia, it is frequently used as a complementary treatment or health supplement, while in western countries, weight losing is an important purpose (Chen and Fontana, 2021). Therefore, in order to draw attention and provide research directions, it is important to evaluate current research trends.

The number of publications on CHM’s toxicity increased in the past decade. The top 5 journals with the most publications have a great influence, with half of the highly cited publications (5/10) included. The *Journal of Ethnopharmacology* was considered the most influential journal within the field, exhibiting the highest publication and citation frequencies. Although most studies were funded and completed by Chinese institutions and authors, publications were concentrated in non-native journals. This highlights the need for more high-quality local journals to accommodate the growing studies. Similar to other study on toxicity (He et al., 2022), this study found that 9 of the top 10 cited studies were reviews, with only 1 being original research. While reviews contribute to summarizing research progress, this phenomenon may indicate a lack of attention to original research.

In keywords, this study identified hepatotoxicity and nephrotoxicity as the most concerned toxicity categories associated with CHM. Hepatotoxicity is the most common toxicity observed, with over 40 CHMs being identified as potential causes of liver injury (Teschke et al., 2014; Frenzel and Teschke, 2016). Among these toxic substances, alkaloids and terpenoids represent the two primary groups associated with hepatotoxic effects (He et al., 2019). Nephrotoxic components in CHM, particularly aristolochic acid, were among the earliest lethal toxic components identified in CHM. Besides, additional components that cause nephrotoxicity include alkaloids and anthraquinones. Although certain toxicities related to CHM were reported, including neurotoxicity (e.g., *Tripterygium wilfordi* (Liu et al., 2019)), cardiotoxicity (e.g., *Aconitum carmichaeli* (Sun et al., 2018)), and reproductive toxicity (e.g., *Rhizoma Pinelliae* (Li et al., 2022)), these do not exhibit high keyword frequency. This could be attributed to the fact that many CHMs possess a multitoxic nature, including hepatotoxicity or nephrotoxicity. Apoptosis was the most concerned pathological mechanism, which is quite complex. Toxic components such as triptolide and aristolochic acid can induce apoptosis to cause damage (Romanov et al., 2015; Wang Y. et al., 2018). Conversely, oxymatrine in *Sophora flavescens* may promote apoptosis to provide anti-cancer effect (Lan et al., 2020). In addition, evidence suggest that emodin in several CHMs can reduce the toxicity of cisplatin by inhibiting apoptosis (Liu et al., 2016). In methodology, the diversity reflected in the keywords of high-frequency, including various experimental and statistical methods. Regarding toxic ingredients, triptolide (n = 53), flavonodis (n = 25) and aristolochic acid (n = 21) keywords used over 20 times each. Expect aristolochic acids, as the most classic toxic ingredient, the other two exhibit dual characteristics. Triptolide functions as both an active and toxic ingredient, showcasing antitumor properties alongside notable hepatotoxic, nephrotoxic, and cardiotoxic tendencies (Noel et al., 2019). Flavonoids exists in most plant species, yet certain members within this category carry nephrotoxic properties (Yang et al., 2018). Other classic toxic components received less attention in keyword mentions, likely attributable to the prohibition of corresponding CHM, consequently diminishing research focus.

Co-occurrence clustering analysis identified 3 clusters related to CHM, mechanism and pharmacology, respectively. Although all included publications involved both CHM and toxicity, the connection between these 2 words and their derivatives were relatively loose. This may be attributed to studies focusing predominantly on one keyword and giving less attention to the other, leading to a low frequency of co-occurrence. Therefore, there remains insufficient studies that directly explore the intersection of CHM and toxicity. Keywords with short-term durations indicate limited research importance in these topics. In long-term keywords, apart from toxicity and safety, angiogenesis and neuroprotection had weak correlation with CHM’s toxicity itself. On the contrary, some low-toxicity CHMs were shown to possess neuroprotective or anti-angiogenic properties, thereby reducing neurotoxicity or cardiotoxicity induced by other drugs (Cheng et al., 2016; Li et al., 2019). These keywords could provide new insights into the relationship between CHM and toxicity. Emerging keywords in the past 3 years included 2 mechanism-related terms and 2 methodological terms. Within the mechanism category, ferroptosis is a non-apoptotic form of cell death, which is also an effective index for monitoring kidney injury (Jiang et al., 2021; Zeng et al., 2023). A recent study indicated that certain CHMs containing arsenic can induce kidney injury through the induction of ferroptosis (Zhang et al., 2022). The NLRP3 inflammasome was recognized as a trigger for liver injury (Mridha et al., 2017). In CHMs, *Epimedium brevicornu* and *Psoralea corylifolia* were proved to cause liver injury by enhancing NLRP3 inflammasome activation (Wang et al., 2020; Qin et al., 2021). Therefore, further studies concerning hepatotoxicity and nephrotoxicity in CHMs could take these 2 topics into consideration. Machine learning and network pharmacology were emerging technologies in the field of pharmacology. In the study of CHM, machine learning exists the potential in predicting toxicity (Zulkifli et al., 2023), while network pharmacology could be utilized to compensate for the limitations of traditional research methods in understanding the multi-component synergism of CHM (Yuan et al., 2017). Although landmark achievements in these 2 methods within CHM toxicity research are currently limited, they hold potential for future directions. It should be emphasized that these methods, based on existing evidence, rely on the accumulation of data from original studies.

One limitation is that the study was conducted on existing publications, which was unable to represent undiscovered research directions. Despite this common shortcoming of this type of research, this study attempted to explore future research directions by summarizing emerging keywords. Another limitation is that the influence of a journal or individual publication may not be fully consistent with the number of citations received. Aside from publication time and exposure, citation rate may even be affected by the popularity of the publisher or author (Hirsch, 2005). To mitigate this limitation, this study included only publications from the last 10 years to avoid diluting recent trends with outdated studies or methods. Additionally, potential bias may arise from the constraint of current software in merging database analyses. In order to mitigate this source of error, WOS was designated as the principal database for analysis, with supplementary examination of the CNKI database aimed at exploring divergences between the two databases.

## 5 Conclusion

Concerns about the toxicity of CHM have increased in the past decade. However, there remains insufficient studies that directly explore the intersection of CHM and toxicity. Hepatotoxicity and nephrotoxicity, as the most concerned toxicity classifications associated with CHM, warrant more in-depth investigations. Apoptosis was the most concerned toxicological mechanism. As a recent increase in attention, exploring the mechanisms of ferroptosis in nephrotoxicity and NLRP3 inflammasome in hepatotoxicity could provide valuable insights. Machine learning and network pharmacology are potential methods for future studies.

## Data Availability

The original contributions presented in the study are included in the article/Supplementary Material, further inquiries can be directed to the corresponding authors.

## References

[B1] Barnes J. McLachlan A. J. Sherwin C. M. Enioutina E. Y. (2016). Herbal medicines: challenges in the modern world. Part 1. Australia and New Zealand. Expert Rev. Clin. Pharmacol. 9, 905–915. 10.1586/17512433.2016.1171712 27070431

[B2] Chen V. L. Fontana R. J. (2021). Are herbals more hepatotoxic than prescription medications. Hepatol. Int. 15, 1301–1304. 10.1007/s12072-021-10256-w 34609679 PMC10184055

[B3] Cheng J. H. Tsai C. L. Lien Y. Y. Lee M. S. Sheu S. C. (2016). High molecular weight of polysaccharides from Hericium erinaceus against amyloid beta-induced neurotoxicity. BMC Complement. Altern. Med. 16, 170. 10.1186/s12906-016-1154-5 27266872 PMC4895996

[B4] Donaldson L. J. Kelley E. T. Dhingra-Kumar N. Kieny M. P. Sheikh A. (2017). Medication without harm: WHO's third global patient safety challenge. Lancet 389, 1680–1681. 10.1016/S0140-6736(17)31047-4 28463129

[B5] Ekor M. (2014). The growing use of herbal medicines: issues relating to adverse reactions and challenges in monitoring safety. Front. Pharmacol. 4, 177. 10.3389/fphar.2013.00177 24454289 PMC3887317

[B6] Frenzel C. Teschke R. (2016). Herbal hepatotoxicity: clinical characteristics and listing compilation. Int. J. Mol. Sci. 17, 588. 10.3390/ijms17050588 27128912 PMC4881436

[B7] Gabardi S. Munz K. Ulbricht C. (2007). A review of dietary supplement-induced renal dysfunction. Clin. J. Am. Soc. Nephrol. 2, 757–765. 10.2215/CJN.00500107 17699493

[B8] Hao P. Jiang F. Cheng J. Ma L. Zhang Y. Zhao Y. (2017). Traditional Chinese medicine for cardiovascular disease: evidence and potential mechanisms. J. Am. Coll. Cardiol. 69, 2952–2966. 10.1016/j.jacc.2017.04.041 28619197

[B9] Hashimoto K. Higuchi M. Makino B. Sakakibara I. Kubo M. Komatsu Y. (1999). Quantitative analysis of aristolochic acids, toxic compounds, contained in some medicinal plants. J. Ethnopharmacol. 64, 185–189. 10.1016/s0378-8741(98)00123-8 10197755

[B10] He S. Zhang C. Zhou P. Zhang X. Ye T. Wang R. (2019). Herb-induced liver injury: phylogenetic relationship, structure-toxicity relationship, and herb-ingredient network analysis. Int. J. Mol. Sci. 20, 3633. 10.3390/ijms20153633 31349548 PMC6695972

[B11] He T. Ao J. Duan C. Yan R. Li X. Liu L. (2022). Bibliometric and visual analysis of nephrotoxicity research worldwide. Front. Pharmacol. 13, 940791. 10.3389/fphar.2022.940791 36188597 PMC9515790

[B12] Hirsch J. E. (2005). An index to quantify an individual's scientific research output. Proc. Natl. Acad. Sci. U.S.A. 102, 16569–16572. 10.1073/pnas.0507655102 16275915 PMC1283832

[B13] Hoppmann N. A. Gray M. E. McGuire B. M. (2020). Drug-induced liver injury in the setting of chronic liver disease. Clin. Liver Dis. 24, 89–106. 10.1016/j.cld.2019.09.006 31753253

[B14] Jakobsson P. J. Robertson L. Welzel J. Zhang M. Zhihua Y. Kaixin G. (2022). Where traditional Chinese medicine meets Western medicine in the prevention of rheumatoid arthritis. J. Intern. Med. 292, 745–763. 10.1111/joim.13537 35854675 PMC9796271

[B15] Jiang X. Stockwell B. R. Conrad M. (2021). Ferroptosis: mechanisms, biology and role in disease. Nat. Rev. Mol. Cell Biol. 22, 266–282. 10.1038/s41580-020-00324-8 33495651 PMC8142022

[B16] Kulkarni P. (2021). Prediction of drug-induced kidney injury in drug discovery. Drug Metab. Rev. 53, 234–244. 10.1080/03602532.2021.1922436 34000943

[B17] Lan X. Zhao J. Zhang Y. Chen Y. Liu Y. Xu F. (2020). Oxymatrine exerts organ- and tissue-protective effects by regulating inflammation, oxidative stress, apoptosis, and fibrosis: from bench to bedside. Pharmacol. Res. 151, 104541. 10.1016/j.phrs.2019.104541 31733326

[B18] Lee T. Y. Wu M. L. Deng J. F. Hwang D. F. (2002). High-performance liquid chromatographic determination for aristolochic acid in medicinal plants and slimming products. J. Chromatogr. B Anal. Technol. Biomed. Life Sci. 766, 169–174. 10.1016/s0378-4347(01)00416-9 11820292

[B19] Li C. Niu M. Bai Z. Zhang C. Zhao Y. Li R. (2017). Screening for main components associated with the idiosyncratic hepatotoxicity of a tonic herb, Polygonum multiflorum. Front. Med. 11, 253–265. 10.1007/s11684-017-0508-9 28315126

[B20] Li J. Wu Y. Wang D. Zou L. Fu C. Zhang J. (2019). Oridonin synergistically enhances the anti-tumor efficacy of doxorubicin against aggressive breast cancer via pro-apoptotic and anti-angiogenic effects. Pharmacol. Res. 146, 104313. 10.1016/j.phrs.2019.104313 31202781

[B21] Li Q. Yan X. Zhang Y. Zhou J. Yang L. Wu S. (2022). Risk compounds, potential mechanisms and biomarkers of traditional Chinese medicine-induced reproductive toxicity. J. Appl. Toxicol. 42, 1734–1756. 10.1002/jat.4290 35075663

[B22] Liu C. Zhang C. Wang W. Yuan F. He T. Chen Y. (2019). Integrated metabolomics and network toxicology to reveal molecular mechanism of celastrol induced cardiotoxicity. Toxicol. Appl. Pharmacol. 383, 114785. 10.1016/j.taap.2019.114785 31629732

[B23] Liu H. Gu L. B. Tu Y. Hu H. Huang Y. R. Sun W. (2016). Emodin ameliorates cisplatin-induced apoptosis of rat renal tubular cells *in vitro* by activating autophagy. Acta Pharmacol. Sin. 37, 235–245. 10.1038/aps.2015.114 26775661 PMC4753365

[B24] Liu S. H. Chuang W. C. Lam W. Jiang Z. Cheng Y. C. (2015). Safety surveillance of traditional Chinese medicine: current and future. Drug Saf. 38, 117–128. 10.1007/s40264-014-0250-z 25647717 PMC4348117

[B25] Liu Y. Wang Q. Yang J. Guo X. Liu W. Ma S. (2018). Polygonum multiflorum Thunb.: a review on chemical analysis, processing mechanism, quality evaluation, and hepatotoxicity. Front. Pharmacol. 9, 364. 10.3389/fphar.2018.00364 29713283 PMC5912012

[B26] Lv W. Piao J. H. Jiang J. G. (2012). Typical toxic components in traditional Chinese medicine. Expert Opin. Drug Saf. 11, 985–1002. 10.1517/14740338.2012.726610 22992190

[B27] Ma Z. T. Shi Z. Xiao X. H. Wang J. B. (2023). New insights into herb-induced liver injury. Antioxid. Redox Signal. 38, 1138–1149. 10.1089/ars.2022.0134 36401515 PMC10259609

[B28] Mridha A. R. Wree A. Robertson A. Yeh M. M. Johnson C. D. Van Rooyen D. M. (2017). NLRP3 inflammasome blockade reduces liver inflammation and fibrosis in experimental NASH in mice. J. Hepatol. 66, 1037–1046. 10.1016/j.jhep.2017.01.022 28167322 PMC6536116

[B29] Noel P. Von Hoff D. D. Saluja A. K. Velagapudi M. Borazanci E. Han H. (2019). Triptolide and its derivatives as cancer therapies. Trends Pharmacol. Sci. 40, 327–341. 10.1016/j.tips.2019.03.002 30975442

[B30] Qin N. Xu G. Wang Y. Zhan X. Gao Y. Wang Z. (2021). Bavachin enhances NLRP3 inflammasome activation induced by ATP or nigericin and causes idiosyncratic hepatotoxicity. Front. Med. 15, 594–607. 10.1007/s11684-020-0809-2 33909257

[B31] Qu L. Li X. Xiong Y. Wang Z. Zhou Y. Zou W. (2022). Opportunities and hurdles to European market access for multi-herbal traditional Chinese medicine products: an analysis of EU regulations for combination herbal medicinal products. Pharmacol. Res. 186, 106528. 10.1016/j.phrs.2022.106528 36332812

[B32] Raynor D. K. Dickinson R. Knapp P. Long A. F. Nicolson D. J. (2011). Buyer beware? Does the information provided with herbal products available over the counter enable safe use. BMC Med. 9, 94. 10.1186/1741-7015-9-94 21827684 PMC3180693

[B33] Romanov V. Whyard T. C. Waltzer W. C. Grollman A. P. Rosenquist T. (2015). Aristolochic acid-induced apoptosis and G2 cell cycle arrest depends on ROS generation and MAP kinases activation. Arch. Toxicol. 89, 47–56. 10.1007/s00204-014-1249-z 24792323

[B34] Shen T. Liu Y. Shang J. Xie Q. Li J. Yan M. (2019). Incidence and Etiology of drug-induced liver injury in mainland China. Gastroenterology 156, 2230–2241. 10.1053/j.gastro.2019.02.002 30742832

[B35] Sun W. Yan B. Wang R. Liu F. Hu Z. Zhou L. (2018). *In vivo* acute toxicity of detoxified Fuzi (lateral root of Aconitum carmichaeli) after a traditional detoxification process. EXCLI J. 17, 889–899. 10.17179/excli2018-1607 30564068 PMC6295630

[B36] Teng L. Zu Q. Li G. Yu T. Job K. M. Yang X. (2016). Herbal medicines: challenges in the modern world. Part 3. China and Japan. Expert Rev. Clin. Pharmacol. 9, 1225–1233. 10.1080/17512433.2016.1195263 27232545

[B37] Teschke R. Wolff A. Frenzel C. Schulze J. (2014). Review article: herbal hepatotoxicity--an update on traditional Chinese medicine preparations. Aliment. Pharmacol. Ther. 40, 32–50. 10.1111/apt.12798 24844799

[B38] Tu Y. (2016). Artemisinin-A gift from traditional Chinese medicine to the world (Nobel lecture). Angewandte Chemie Int. ed. Engl. 55, 10210–10226. 10.1002/anie.201601967 27488942

[B39] van Eck N. J. Waltman L. (2010). Software survey: VOSviewer, a computer program for bibliometric mapping. Scientometrics 84, 523–538. 10.1007/s11192-009-0146-3 20585380 PMC2883932

[B40] Vanhaelen M. Vanhaelen-Fastre R. But P. Vanherweghem J. L. (1994). Identification of aristolochic acid in Chinese herbs. Lancet 343, 174. 10.1016/s0140-6736(94)90964-4 7904018

[B41] Wang J. Song H. Ge F. Xiong P. Jing J. He T. (2022). Landscape of DILI-related adverse drug reaction in China Mainland. Acta Pharm. Sin. B 12, 4424–4431. 10.1016/j.apsb.2022.04.019 36561993 PMC9764066

[B42] Wang L. Ding X. Li C. Zhao Y. Yu C. Yi Y. (2018a). Oral administration of Aristolochia manshuriensis Kom in rats induces tumors in multiple organs. J. Ethnopharmacol. 225, 81–89. 10.1016/j.jep.2018.07.001 30008395

[B43] Wang Y. Guo S. H. Shang X. J. Yu L. S. Zhu J. W. Zhao A. (2018b). Triptolide induces Sertoli cell apoptosis in mice via ROS/JNK-dependent activation of the mitochondrial pathway and inhibition of Nrf2-mediated antioxidant response. Acta Pharmacol. Sin. 39, 311–327. 10.1038/aps.2017.95 28905938 PMC5800476

[B44] Wang Z. Xu G. Wang H. Zhan X. Gao Y. Chen N. (2020). Icariside Ⅱ, a main compound in Epimedii Folium, induces idiosyncratic hepatotoxicity by enhancing NLRP3 inflammasome activation. Acta Pharm. Sin. B 10, 1619–1633. 10.1016/j.apsb.2020.03.006 33088683 PMC7564030

[B45] Yang B. Xie Y. Guo M. Rosner M. H. Yang H. Ronco C. (2018). Nephrotoxicity and Chinese herbal medicine. Clin. J. Am. Soc. Nephrol. 13, 1605–1611. 10.2215/CJN.11571017 29615394 PMC6218812

[B46] Yang Y. Ge F. L. Tang J. F. Qin S. L. Zeng R. Yao M. L. (2022). A review of herb-induced liver injury in mainland China. Front. Pharmacol. 13, 813073. 10.3389/fphar.2022.813073 36304164 PMC9592926

[B47] Yao C. L. Zhang J. Q. Li J. Y. Wei W. L. Wu S. F. Guo D. A. (2021). Traditional Chinese medicine (TCM) as a source of new anticancer drugs. Nat. Prod. Rep. 38, 1618–1633. 10.1039/d0np00057d 33511969

[B48] You L. Liang K. An R. Wang X. (2022). The path towards FDA approval: a challenging journey for Traditional Chinese Medicine. Pharmacol. Res. 182, 106314. 10.1016/j.phrs.2022.106314 35718244

[B49] Yuan H. Ma Q. Cui H. Liu G. Zhao X. Li W. (2017). How can synergism of traditional medicines benefit from network pharmacology. Molecules 22, 1135. 10.3390/molecules22071135 28686181 PMC6152294

[B50] Zeng F. Nijiati S. Liu Y. Yang Q. Liu X. Zhang Q. (2023). Ferroptosis MRI for early detection of anticancer drug-induced acute cardiac/kidney injuries. Sci. Adv. 9, eadd8539. 10.1126/sciadv.add8539 36888714 PMC9995079

[B51] Zhang S. Cao S. Zhou H. Li L. Hu Q. Mao X. (2022). Realgar-induced nephrotoxicity via ferroptosis in mice. J. Appl. Toxicol. 42, 1843–1853. 10.1002/jat.4362 35803278

[B52] Zhong Y. Menon M. C. Deng Y. Chen Y. He J. C. (2015). Recent advances in traditional Chinese medicine for kidney disease. Am. J. Kidney Dis. 66, 513–522. 10.1053/j.ajkd.2015.04.013 26015275

[B53] Zhou J. Ouedraogo M. Qu F. Duez P. (2013). Potential genotoxicity of traditional Chinese medicinal plants and phytochemicals: an overview. Phytother. Res. 27, 1745–1755. 10.1002/ptr.4942 23420770

[B54] Zulkifli M. H. Abdullah Z. L. Mohamed Yusof N. Mohd Fauzi F. (2023). *In silico* toxicity studies of traditional Chinese herbal medicine: a mini review. Curr. Opin. Struct. Biol. 80, 102588. 10.1016/j.sbi.2023.102588 37028096

