# Supplementary material for: Growing attention on the toxicity of Chinese herbal medicine: a bibliometric analysis from 2013 to 2022
Source: Front Pharmacol. 2024 Feb 1;15:1293468. doi: 10.3389/fphar.2024.1293468 (PMC10867220; doi:10.3389/fphar.2024.1293468)
Supplement: Supplementary file 1 [file DataSheet1.PDF]

Figure S1. Co-occurrence analysis on the 40 keywords of the most records in CNKI

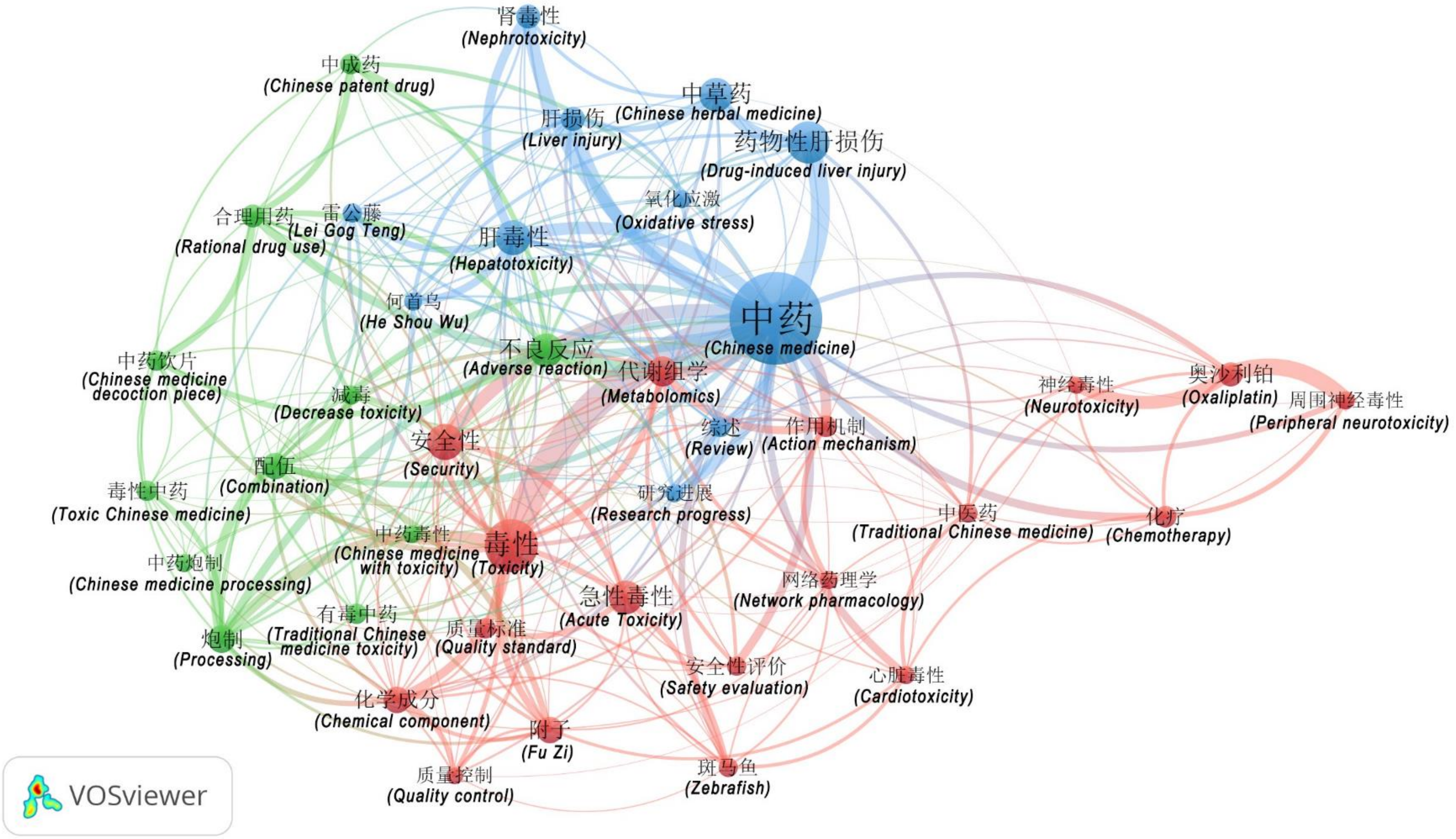

Table S1. Top 40 keywords with the most frequencies in CNKI

Keyword	Count	Keyword	Count
Chinese medicine	654	Chinese medicine decoction piece	81
Toxicity	305	Toxic Chinese medicine	81
Drug-induced liver injury	231	Quality standard	79
Security	187	Chinese patent drug	78
Hepatotoxicity	176	Chinese medicine with toxicity	77
Acute toxicity	166	Decrease toxicity	75
Adverse reaction	165	Lei Gong Teng	74
Chinese herbal medicine	161	Traditional Chinese medicine	74
Metabolomics	139	Zebrafish	69
Processing	126	Safety evaluation	69
Fu Zi	116	He Shou Wu	68
Chemical component	115	Cardiotoxicity	68
Combination	115	Network pharmacology	66
Oxaliplatin	99	Traditional Chinese medicine toxicity	66
Nephrotoxicity	98	Peripheral neurotoxicity	65
Liver injury	97	Chinese medicine processing	65
Rational drug use	96	Oxidative stress	64
Action mechanism	84	Research progress	63
Chemotherapy	84	Quality control	62
Review	82	Neurotoxicity	59

Supplementary method

Search strategy for the CNKI database:
#1 Theme = “toxicity (毒性）” OR “toxicology (毒理学）” OR “nephrotoxicity (肾毒性）” OR “hepatotoxicity（肝毒性）” OR “neurotoxicity（神经毒性）” OR “cardiotoxicity (心脏毒性）” OR “ototoxicity (耳毒性）” OR “hematotoxicity (血液毒性）” OR “immunotoxicity (免疫毒性）” OR “drug-induced injury (药物性损伤）”
#2 Theme = “Chinese medicine (中药）” OR “Chinese herb (中草药)”
#3 Publication Time = 2013-2022
#1 AND #2 AND #3
